# *Caveat emptor*: the combined effects of multiplicity and selective reporting

**DOI:** 10.1186/s13063-018-2888-9

**Published:** 2018-09-17

**Authors:** Tianjing Li, Evan Mayo-Wilson, Nicole Fusco, Hwanhee Hong, Kay Dickersin

**Affiliations:** 1 Department of Epidemiology, Johns Hopkins University Bloomberg School of Public Health, 615 North Wolfe Street, Baltimore, MD 21205 USA; 2 Department of Biostatistics and Bioinformatics, Duke University School of Medicine, 2424 Erwin Road, Suite 1105, 11041 Hock Plaza, Durham, NC 27705 USA

**Keywords:** Multiplicity, Selective reporting, Reproducibility

## Abstract

Clinical trials and systematic reviews of clinical trials inform healthcare decisions. There is growing concern, however, about results from clinical trials that cannot be reproduced. Reasons for nonreproducibility include that outcomes are defined in multiple ways, results can be obtained using multiple methods of analysis, and trial findings are reported in multiple sources (“multiplicity”). Multiplicity combined with selective reporting can influence dissemination of trial findings and decision-making. In particular, users of evidence might be misled by exposure to selected sources and overly optimistic representations of intervention effects. In this commentary, drawing from our experience in the Multiple Data Sources in Systematic Reviews (MUDS) study and evidence from previous research, we offer practical recommendations to enhance the reproducibility of clinical trials and systematic reviews.

## Background

Clinical trials and systematic reviews of clinical trials inform healthcare decisions, but there is growing concern that the methods and results of some clinical trials are not reproducible [1]. Poor design, careless execution, and variation in reporting contribute to nonreproducibility [2, 3]. In addition, trials may not be reproducible because trialists have reported their studies selectively [4]. Although steps now being taken toward “open science” are designed to enhance reproducibility [5, 6], such as trial registration and mandatory results reporting [7–13], making trial protocols and results public may lead to a glut of data and sources that few scientists have the resources to explore. This well-needed approach will thus not serve as a panacea for the problem of nonreproducibility.

Goodman and colleagues argue that “multiplicity, combined with incomplete reporting, might be the single largest contributor to the phenomenon of nonreproducibility, or falsity, of published claims [in clinical research]” ([14], p. 4). We define multiplicity in clinical research to include assessing multiple outcomes, using multiple statistical models, and reporting in multiple sources. When multiplicity is used by investigators to selectively report trial design and findings, misleading information is transmitted to evidence users.

Multiplicity was evident in a study we recently conducted, the Multiple Data Sources in Systematic Reviews (MUDS) project [15–19]. In this paper, drawing from our experience in the MUDS study and evidence from previous research, we offer practical recommendations to enhance the reproducibility of clinical trials and systematic reviews.

### Multiplicity of outcomes

Choosing appropriate outcomes is a critical step in designing valid and useful clinical trials. An outcome is an event following an intervention that is used to assess its safety and/or efficacy [20]. For randomized controlled trials (RCTs), outcomes should be clinically relevant and important to patients, and they should capture the causal effects of interventions; core outcome sets aim to do this [21].

A clear outcome definition includes the domain (e.g., pain), the specific measurement tool or instrument (e.g., short form of the McGill Pain Questionnaire), the time point of assessment (e.g., 8 weeks), the specific metric used to characterize each participant’s results (e.g., change from baseline to a specific time point), and the method of aggregating data within each group (e.g., mean) (Table 1) [22, 23]. Multiplicity in outcomes occurs when, for one outcome “domain,” there are variations in the other four elements [17]. For example, a trial can collect data on many outcomes under the rubric of “pain,” introducing multiplicity and the possibility for selectively reporting a pain outcome associated with the most favorable results. Likewise, a systematic review may specify only the outcome domain, allowing for variations in all other elements [24].

**Table 1:** Elements needed to define an outcome

Element	Description
1. Domain	Title or concept that describes the outcome
2. Specific measure	Tool or instrument that assesses the outcome domain, including the name of the tool or instrument and/or specific diagnostic criteria and ascertainment procedures
3. Time point	When the outcome will be assessed
4. Specific metric	Ways to characterize measurement on each individual (e.g., change in a measurement from baseline to a specific time point)
5. Method of aggregation	Ways to summarize individual-level measurements into group-level statistics for estimating treatment effect, including if the outcome will be treated as a continuous, categorical, or time-to-event variable and, if relevant, the specific cutoff or categories

To illustrate how “cherry-picking” an outcome can work, in a Pfizer study that compared celecoxib with placebo in osteoarthritis of the knee, the investigators noted, “The WOMAC [Western Ontario and McMaster Universities] pain subscale was the most responsive of all five pain measures. Pain–activity composites resulted in a statistically significant difference between celecoxib and placebo but were not more responsive than pain measures alone. However, a composite responder defined as having 20% improvement in pain or 10% improvement in activity yielded much larger differences between celecoxib and placebo than with pain scores alone” ([25], p. 247).

### Multiplicity of analyses and results

The goal of the analysis in an RCT is to draw inferences regarding the intervention effect by contrasting group-level quantities. Numerical contrasts between groups, which are typically ratios (e.g., relative risk) or differences in values (e.g., difference in means), are the results of the trial. There are numerous ways to analyze data for a defined outcome; thus, multiple methods of analysis introduce another dimension of multiplicity in clinical trials [17]. For example, one could analyze data on all or a subset of the participants, use multiple methods for handling missing data, and adjust for different covariates.

Although it makes sense that a range of analyses may be conducted to ensure that the findings are robust to different assumptions made about the data, performing analysis multiple ways and obtaining different results can lead to selective reporting of results deemed as favorable by the study investigators [26, 27].

### Multiplicity of sources

Trial results can be reported in multiple places. This creates problems for users because many sources present incomplete or unclear information, and by reporting in multiple sources, investigators may present conflicting results. When we compared all data sources for trials included in the MUDS project, we found that information about trial characteristics and risk of bias often differed across reports, and conflicting information was difficult to disentangle [18]. In addition, important information about certain outcomes was available only in nonpublic sources [17]. Additionally, information within databases may change over time. In trial registries, outcomes may be changed, deleted, or added; although changes are documented in the archives of ClinicalTrials.gov, they are easily overlooked.

### The consequences of multiplicity in RCTs

Compared with the number of “domains” in a trial, multiplicity in outcome definitions and methods of analysis may lead to an exponentially larger number of RCT results. This, combined with multiple sources of RCT information, leads to challenges for subsequent evidence synthesis [17].

There are many ways that multiplicity leads to research waste. Arguably, the most prominent example is that when one uses inconsistent outcome definitions across RCTs, trial findings cannot be combined in systematic reviews and meta-analyses even when the individual trials studied the same question [28, 29].

Aggregating results from trials depends on consistency in both outcome domains and the other four elements. Failure to synthesize the quantitative evidence means that health policy, practice guidelines, and healthcare decision-making are not informed by RCT evidence, even though RCTs exist [2, 30, 31]. For example, in a Cochrane eyes and vision systematic review and meta-analysis of RCTs examining methods to control inflammation after cataract surgery, 48 trials were eligible for inclusion in the review. However, no trial contributed to the meta-analysis, because the outcome domain “inflammation” was assessed and aggregated inconsistently [32, 33].

Multiplicity combined with selective reporting can mislead decision-making. There is ample evidence that outcomes associated with positive or statistically significant results are more likely to be reported than outcomes associated with negative or null results [4, 34]. Selective reporting can have three types of consequence for a systematic review: (1) a systematic review may fail to locate an entire trial because it remains unpublished (potential for publication bias); (2) a systematic review may locate the trial but fail to locate all outcomes assessed in the trial (potential for bias in selective reporting of outcomes); and (3) a systematic review may locate all outcomes but fail to locate all numerical results (potential for bias in selective reporting of results) [35]. Any three types of selective reporting threaten the reproducibility of clinical trials and the validity of systematic reviews because they lead to overly optimistic representations of intervention effects. To improve the reproducibility of clinical trials and systematic reviews, we have the recommendations outlined below for trialists and systematic reviewers.

#### Recommendation 1: Trialists should define outcomes using the five-element framework and use core outcome sets whenever possible

Many trials do not define their outcomes completely [23]; yet, simply naming an outcome domain for a trial is insufficient to limit multiplicity, and it invites selective reporting. When outcomes are defined solely in terms of their domains, there is much room for making up multiple outcomes post hoc and cherry-picking favorable results.

In MUDS, we collected data from 21 trials of gabapentin for neuropathic pain. By searching for all sources of information about the trials, we identified 74 reports that described the trial results, including journal articles, conference abstracts, trial registrations, approval packages from the U.S. Food and Drug Administration, and clinical study reports. We also acquired six databases containing individual participant data. For the single outcome domain “pain intensity,” we identified 8 specific measurements (e.g., short form of the McGill Pain Questionnaire, visual analogue scale), 2 specific metrics, and 39 methods of aggregation for an 8-week time window. This resulted in 119 defined outcomes.

#### Recommendation 2: Trialists should produce and update, as needed, a dated statistical analysis plan (SAP) and communicate the plan to the public

It is possible to obtain multiple results for a single outcome by using different methods of analysis [17, 36]. In MUDS, using gabapentin for neuropathic pain as an example, we identified 4 analysis populations and 5 ways of handling missing data from 21 trials, leading to 287 results for pain intensity at an 8-week time window.

We recommend that trialists should produce a SAP before the first participant is randomized, following the recommended guidelines [37]. The International Conference on Harmonisation defines a SAP as “a document that contains a more technical and detailed elaboration of the principal features of the analysis than those described in the protocol, and includes detailed procedures for executing the statistical analysis of the primary and secondary variables and other data” ([38], p. 35). Currently, SAPs are usually prepared for industry-sponsored trials; however, in our opinion, SAPs may not be prepared with the same level of detail for non-industry-sponsored trials [39]. Others have shown a diverse practice with regard to SAP content [37]. The National Institutes of Health has a less specific but similar policy, which became effective January 25, 2018 [40].

It is entirely possible that additional analyses might be conducted after the SAP is drafted, such as at the behest of peer reviewers or a data monitoring committee. In cases such as this, investigators should document and date any amendments to the SAP or protocol and communicate post hoc analyses clearly. SAPs should be made publicly available and linked to other trial information (*see* Recommendation 3).

#### Recommendation 3: Trialists should make information about trial methods and results public, provide references to the information sources in a trial registry, and keep the list of sources up-to-date

Trial methods and results should be made public so that users can assess the validity of the trial findings. Users of trial information should anticipate that there may be multiple sources associated with a trial. A central indexing system, such as a trial registry, for listing all trial information sources should be available so that systematic reviewers can find multiple sources without unnecessary expenditure of resources.

#### Recommendation 4: Systematic reviewers should anticipate a multiplicity of outcomes, results, and sources for trials included in systematic reviews and should describe how they will handle such issues before initiating their research

Systematic reviewers sometimes use explicit rules for data extraction and analysis. For example, some systematic reviewers extract outcomes that were measured using the most common scale or instrument for a particular domain. Although such approaches may be reproducible and efficient, they may exclude data that users consider informative. When rules for selecting from among multiple outcomes and results are not prespecified, the choice of data for meta-analysis may be arbitrary or data-driven. In the MUDS example, if we were to pick all possible combinations of the three elements (specific measure, specific metric, and method of aggregation) for a single outcome domain, pain intensity at an 8-week window (i.e., holding domain and time point constant), we could conduct 34 trillion different meta-analyses [18].

Many authoritative sources recommend looking for all sources of information about each trial identified for a systematic review [41, 42]. To investigate whether multiplicity in results and multiplicity in data sources might influence the conclusions of meta-analysis on pain at 8 weeks, we performed a resampling meta-analysis [43] using MUDS data from the 21 trials and 74 sources as follows:

1. In each resampling iteration, we randomly selected one possible result from each trial within a prespecified 8-week time window.
2. We combined the sampled results using a random effects meta-analysis.
3. We iterated the first two steps 10,000 times;
4. We generated a histogram that shows the distribution of the estimates from meta-analyses.

**Fig. 1:**
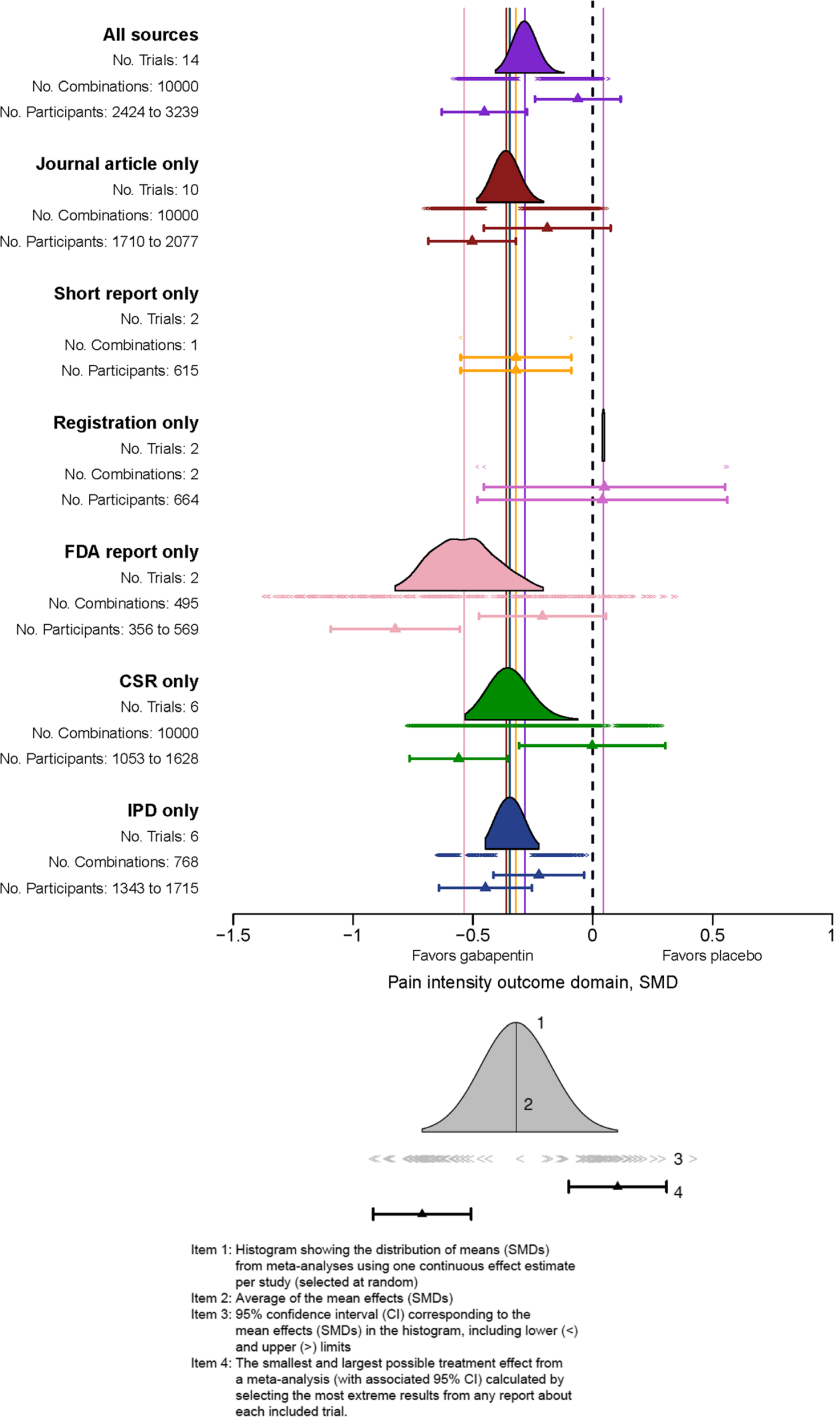
Results of the resampling meta-analyses for pain intensity at 8 weeks [18]. *CSR* Clinical Study Report, *FDA* U.S. Food and Drug Administration, *IPD* Individual patient data, *SMD* Standardized mean difference

As shown in the top histogram of Fig. 1, when all sources of data were used, meta-analyses that included the largest and smallest estimates from each trial could lead to different conclusions on the effectiveness of gabapentin with nonoverlapping 95% CIs. When the resampling meta-analyses were repeated using only one data source at a time, we found that there was variation in the results by data source.

## Conclusions

Multiplicity of outcomes, analyses, results, and sources, coupled with selective reporting, can affect the findings of individual trials as well as the systematic reviews and meta-analyses based on them. We encourage trialists and systematic reviewers to consider our recommendations aimed at minimizing the effects of multiplicity on what we know about intervention effectiveness.

## Abbreviations

MUDS: Multiple Data Sources in Systematic Reviews
RCT: Randomized controlled trial
SAP: Statistical analysis plan
